# Association of Cyclin Dependent Kinase 10 and Transcription Factor 2 during Human Corneal Epithelial Wound Healing *in vitro* model

**DOI:** 10.1038/s41598-019-48092-6

**Published:** 2019-08-14

**Authors:** Meraj Zehra, Shamim Mushtaq, Syed Ghulam Musharraf, Rubina Ghani, Nikhat Ahmed

**Affiliations:** 1 Department of Research, Department of Biochemistry, Ziauddin University, Karachi, 75600 Pakistan; 2 Department of Biochemistry, University of Karachi, Karachi, 75500 Pakistan; 3 Dr. Panjwani Center for Molecular Medicine and Drug Research, International Center for Chemical and Biological Sciences, Karachi, Pakistan; 4 H.E.J. Research Institute of Chemistry, International, Center for Chemical and Biological Sciences,University of Karachi, Karachi, 75270 Pakistan; 5 Department of Biochemistry, Jinnah Medical and Dental College, Karachi, 74800 Pakistan; 6 Department of Biochemistry, Barette Hodgson University, Department of Bioscience, Karachi, 74900 Pakistan

**Keywords:** Biomarkers, Cell biology

## Abstract

Proper wound healing is dynamic in order to maintain the corneal integrity and transparency. Impaired or delayed corneal epithelial wound healing is one of the most frequently observed ocular defect and difficult to treat. Cyclin dependen kinase (cdk), a known cell cycle regulator, required for proper proliferating and migration of cell. We therefore investigated the role of cell cycle regulator cdk10, member of cdk family and its functional association with transcriptional factor (ETS2) at active phase of corneal epithelial cell migration. Our data showed that cdk10 was associated with ETS2, while its expression was upregulated at the active phase (18 hours) of cell migration and gradually decrease as the wound was completely closed. Topical treatment with anti-cdk10 and ETS2 antibodies delayed the wound closure time at higest concentration (10 µg/ml) compared to control. Further, our results also showed increased mRNA expression of cdk10 and ETS2 at active phase of migration at approximately 2 fold. Collectively, our data reveals that cdk10 and ETS2 efficiently involved during corneal wound healing. Further studies are warranted to better understand the mechanism and safety of topical cdk10 and ETS2 proteins in corneal epithelial wound-healing and its potential role for human disease treatment.

## Introduction

Corneal epithelial injuries and burns produce extensive damage to the ocular surface epithelium and may cause significant loss of function^1^. A rapid and efficient healing from injuries and environmental damges is necessary to maintain the cornea barrier that is essential for appropriate vision^2^. Delayed in corneal epithelial wounds healing occur in number of disease states, however, persistence of these wounds can lead to loss of vision and even perforation of the eye^3^.

The World Health Organization (WHO) was estimatated 710 corneal ulcers per 100 000 population every year in south east asia region^4^. However, in Pakistan corneal injuries or trauma are the common cause of blindness after cataract but data available about injuries is limited and does not indicate the magnitude of the problem. Recently Baig R *et al*. reported 39.7% eye injuries among all ocular complaints at emergency department (ED) visits in a private tertiary care hospital Karachi Pakistan^5^. In our region, it was also reported that the frequency of trauma was 66% while metallic particles and road accedents were the major cause of ocular injuries^6,7^.

To reduce the potential of these debilitating injury or wounds is to promote the epithelial migration and decrease the chances of ocular infection with limited toxicity. Treatments for non-healing corneal wounds are limited and no specific therapy available so far. Knowing proper cell cycle division mechanism and specifically with their regulators would thus help to development a new therapeutic tools which trigger or control cell migration and proliferation. A fundamental requirement for proper proliferating and migration of cell, is the unidirectional progression of the eukaryotic cell cycle which are ensured by different checkpoints and the oscillating expression of cell cycle proteins.

Cyclin-dependent kinases (cdks), which are belong to a large cell cycle protein family that have been found so far in human cells. In mammalian genome at least 20 different cdks are identified so far and some of them are constant in cell division and play their role to modulate ETS2 transcription factor for its transactivation activity. Cdk10 is a Cdc2-related kinase, which was previously reffered as PISSLRE^8,9^, exerts a positive control on cell division and act as driving regulator during the G2 or M phase of the cell cycle.

However, recent research is reported that cdk10 promotes cell proliferation and regulates transcription and development^10^. However, previous study was also confirmed the cdk10-ETS2 interaction in mammalian cells, suggesting that suppression of ETS2’s transcriptional activity does not depend on the kinase activity of cdk10^11^. Contradictory to this finding, Iorn E *et al*. revealed that cdk10 silences increases ETS2-driven transcription of c-RAF, resulting in mitogen-activated protein kinases (MAPK) pathway activation and loss of tumor cell reliance upon estrogen signaling^12^ but to the best of our knowledge, there is still no study reports the roles of cdk10 and ETS2 during human corneal epithelial cell migration. However, previous studies revealed that cdk5 is predominantly localized along the leading edge of migrating corneal epithelial sheet, and its activity promotes stability of E-cadherin-based cell–cell junctions in human cornea^13–15^. However, for the past twenty years and until recently, the revelation of the functions of cdk10 was hampered by the lack of any identified cdk regulator and its associated partner during corneal epithelial wound healing. In order to gain insight into the control mechanisms of corneal wound healing that determine this proliferative switch, we have investigated the cdk10 expression level and its association with other proteins in these cells.

*In vitro* human corneal wound healing scratch assay provides an outstanding model to examine the proliferative and migration phase of cell cycle and its associated proteins like cdks. For better diagnosis and management of corneal wound healing problems it is important to understand the basic mechanisms that regulate cellular migration and its associated mediators or regulators which are related to dysregulation of cellular proliferation.

The present study examines the expression and association of cdk10 and ETS2 at the active phase of corneal epithelial healing and investigated the possibility that cdk10 and ETS2 interaction may have a role in active phase of corneal healing cells.

## Results

### Human Corneal Epithelial Cell (HCEC) Culture Model

**Figure 1:**
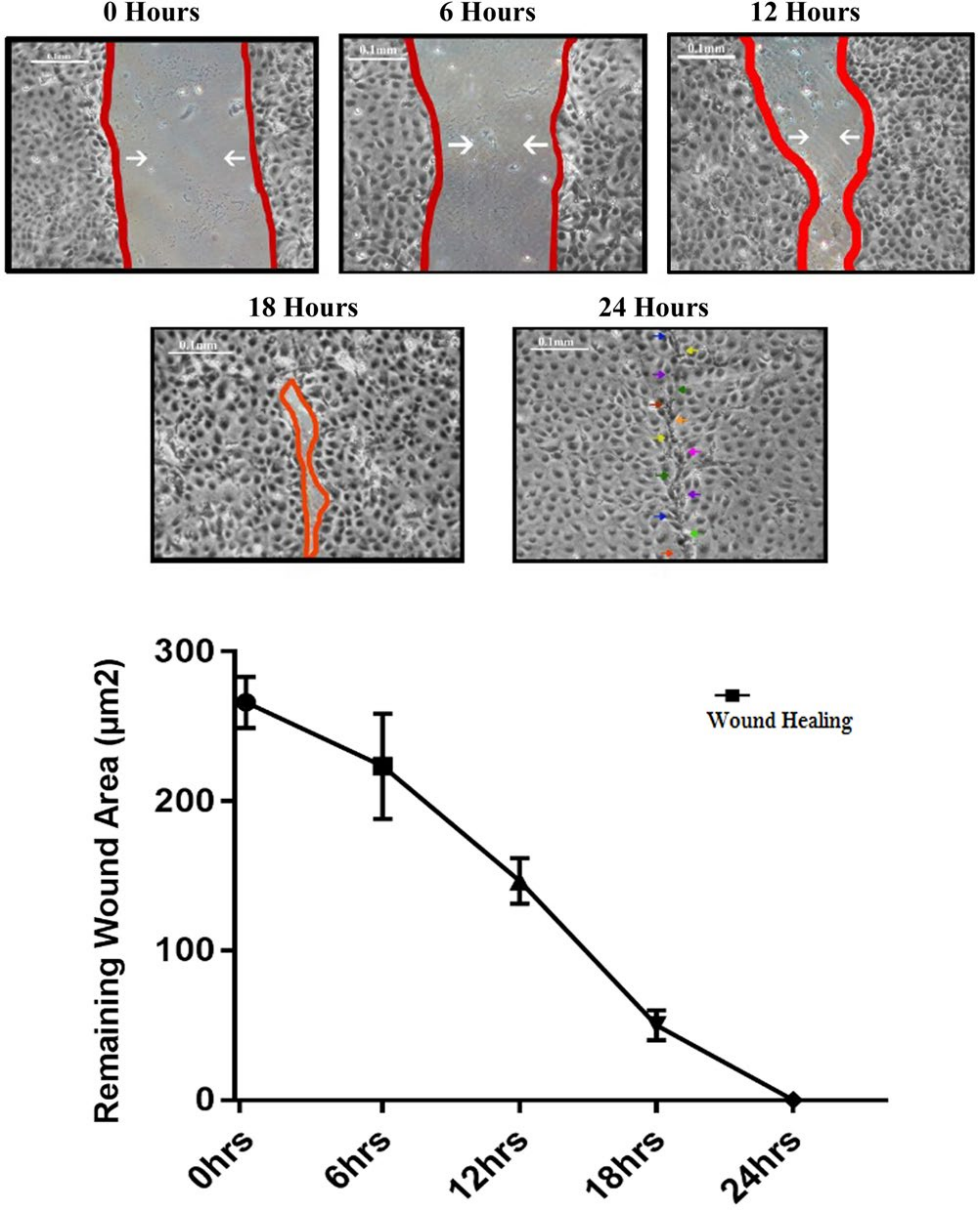
(**A**) Human corneal epithelial cells (HCEC) were subjected to *in vitro* scratch assay. Representative images from scratch wound healing assay of HCEC showing time course of corneal re-epithelialization (abrasion 1 mm) *in vitro* model at different time intervals (0, 6, 12, 18 & 24 hours) after post wounding. Scratch wounds were made in confluent cultures of corneal epithelial cells. The red lines define the area lacking cells where as arrows indicating the movement of cells towards closing the wound. The images were analyzed by Image J software (National Institutes of Health [NIH], Bethesda, MD, USA) with Scale bar = 100 µm. Images were captured at 4X magnification using camera-equipped inverted microscope (Olympus Onvented, DSR-012). (**B**) *In vitro* wound healing of migrating corneal epithelia in confluent monolayer of HCEC showing linear phase of wound healing at time intervals 6, 12, 18 and 24 hours while wound was closed at 24 hours of post wounding. Cellular migration was calculated using one way ANOVA by GraphPad (7.0) with significance of p < 0.001. Each value is representing three individual experiments, error bars indicates SDM.

Corneal epithelial wound healing was evaluated at 0, over a time period (0, 6, 12, 18 & 24 hours) after wounding. The area of wound was measured using Image J software. The average mean area with standard deviation of mean for each time interval was calculated. The extent of healing was defined as the ratio of the differences between the original and the residual wound area. Three different sets of experiments were performed and results were expressed as mean percentage of remaining wound area. At 0 hour when wound was created, the average mean area (µm^2^) was 266 µm^2^ ± 0.017 followed by 230µm^2^ ± 0.0351, 160 µm^2^ ± 0.0152 and 50 µm^2^ ± 0.0011 at 6, 12 and 18 hours respectively (Fig. 1A,B). At 24 hours healing was attained with complete resurfacing of defect (wound closure). The rate of corneal epithelial closure was calculated using linear regression analysis. Table 1 summarizes the healing rate, expressed as µm^2^/hour at different time intervals. The rate of healing during 0–12 hours was 11.6 µm^2^/hours and 0–18 hours was 18.33 µm^2^/hours; r2 = 0.9761; p < 0.001, showing linear phase of migration, hence designated as active phase of wound healing.

**Table 1:** Rate of Human Corneal Epithelial Wound Healing in Cell Culture Model.

Time (hours)	Mean Area (µm^2^)	Rate of healing (µm^2^/hour)	Correlation Coefficient (r2)	
0	266 + 0.0170	Time of debridement	0.9761	*p < 0.001
6	230 + 0.0351	6		
12	160 + 0.0.0152	11.6		
18	50 + 0.0011	18.33		
24	0	Time of Wound closure		

### Two dimensional (2D) Gel Electrophoresis

We were able to analyzed protein differential expressions from human corneal epithelial cells (HCEC) samples (non-migrating and migrating after post wounding (at 6, 12, 18 and 24 hours) by 2DE electrophoresis with the pI range of 3.0–10 and molecular weight range between 20 kDa–200 kDa.

**Figure 2:**
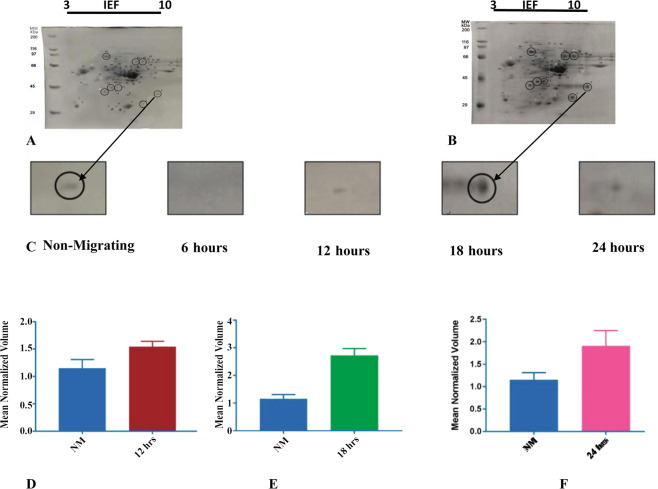
(**A**–**C**) Representative 2D gels of differentially expressed protein at different time course. Protein expressions in (a). Non-migrating and (b). migrating at 18 hours post wounding. **(C**). Protein spot 9 at different time hours and shows gradually decrease expression. (**D**–**F**) Differential Volume of expression of spot 9 at 12, 18 and 24 hours post wounding. The histograms showing normalized protein spot volumes (y-axis) obtained by Melanie 2DE image analysis software 9.0 (Genebio) (2D-gel-analysis.com/melanie-expression). The statistical analysis was performed by *student t-test* using GraphPad prism 7.0 software with p-value < 0.05. Data are presented as the mean ± SD.

The average abundances of differential protein spots were quantified evaluated using Melanie 9.0 software and those with relative changes in abundance greater than 1 times between migrating and non-migrating (upregulated or downregulated) at 95% confidence level (*p* < 0.05) were considered as significant. Representative 2DE gels with loading of an equal amount of total protein (100 µg/gel) revealed approximately 54 protein spots on migrating (active phase of migration at 18 hours) and 49 spots on non-migrating gel (Fig. 2A,B). Minimum numbers of spots (25) were expressed on 6 hours gel exhibiting the latent phase of wound healing whereas 12 hours gel (data not shown) showing gradual increase in protein expression with approximately 40 spots exhibiting linear phase of migration. The 18 hours gel representing the active hours of migration with differential proteins expression selected spots were used for mass spectromentry analysis. The wound was completely healed at 24 hours with gradual decrease in expression of few spots. Epithelial protein spots in all gels were distributed in the following region: isoelectric point (PI) (pH = 3–10) and relative molecular weight (RMW) 200–20 kDa. All gel spots showing significant changes in abundance were highlighted. Most of the protein spots had identical locations (isoelectric point and molecular weight) and similar staining strength between migrating (18 hours) and non-migrating corneal epithelial proteins, whereas some variability was also found. Based on difference in protein intensity between migrating (18 hours) and non- migrating gels eight protein spots were selected for mass spectrometric identification (Data not shown) and in gel digestion as described previously^16^. Current data showed differential expression for only spot 9 at 6, 12, 18 and 24 hours (Fig. 2C–F) for identification.

### Mass Spectrometry

**Figure 3:**
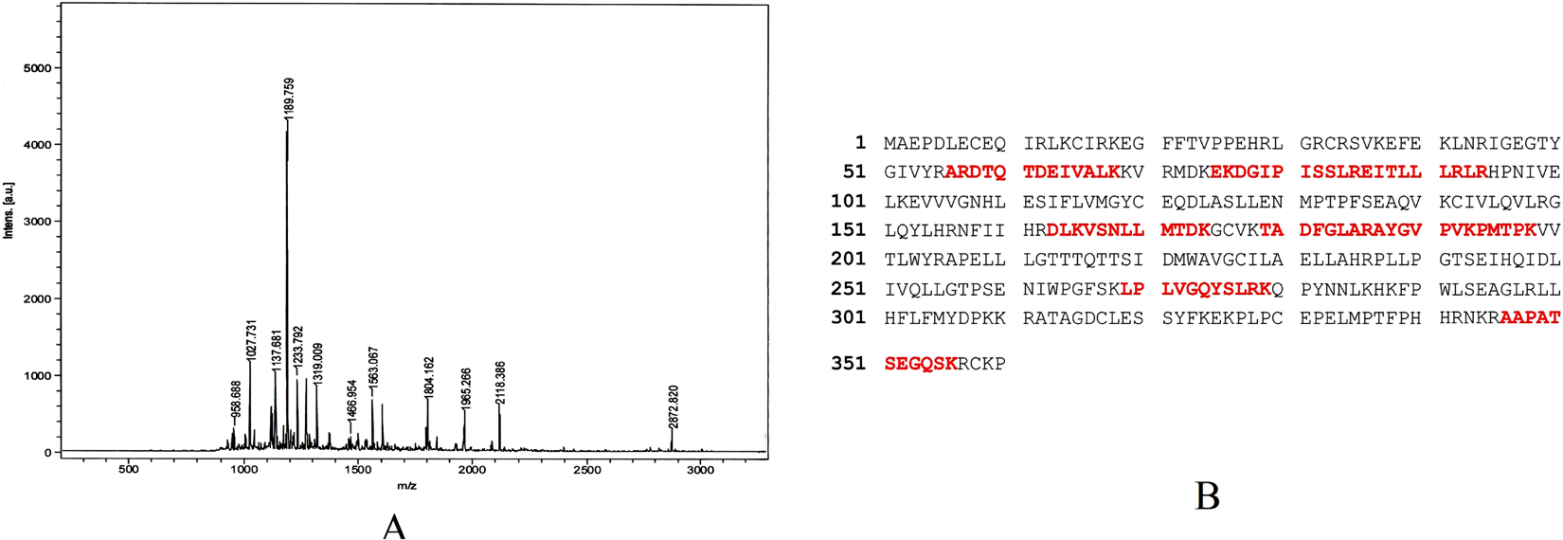
(**A**) MALDI-TOF MS Spectrum of differentially expressed protein spot 9 cdk10. (**B**) The matching rate of peptides with the database are shown in red.

The differentially expressed spot 9 was excised from the 2D gels at 18 hours (Fig. 2C) and subjected to in-gel digestion with trypsin, and the resulting peptides were identified by MALDI-TOF-MS as described previously^16^. Moreover the expression of identified spot 9 protein was consistently increased in migrating sample at 18 hours (Fig. 2C). The MS spectra of above mentioned proteins observed are shown in Fig. 3A. The spot 9 was identified as cyclin dependen kinase 10 (cdk10) from peptide masses and amino acid sequences using MASCOT 2.4 software (Matrix science, London, United Kingdom) against the UniProtKB with *Homo sapiens* species filter (Fig. 3B). Details of the protein spot 9 identifications, protein score, sequence coverage, theoretical pI value and molecular weight as well as average relative change are shown in Table 2.

**Table 2:** Differentially Expressed Proteins cdk10 (spot 9) in HCEC.

Acc #	Name	Abb	Thr.pI/Mass	score	Peptide Matched	% cov	Subcellular Localizat	Biological process	Fold change
Q15131	Cyclin Dependent kinase	cdk10	9.06/41	71	11	22	Cytoskeleton	Catalytic activity	2.37

### The functional association network of identified protein cdk10

**Figure 4:**
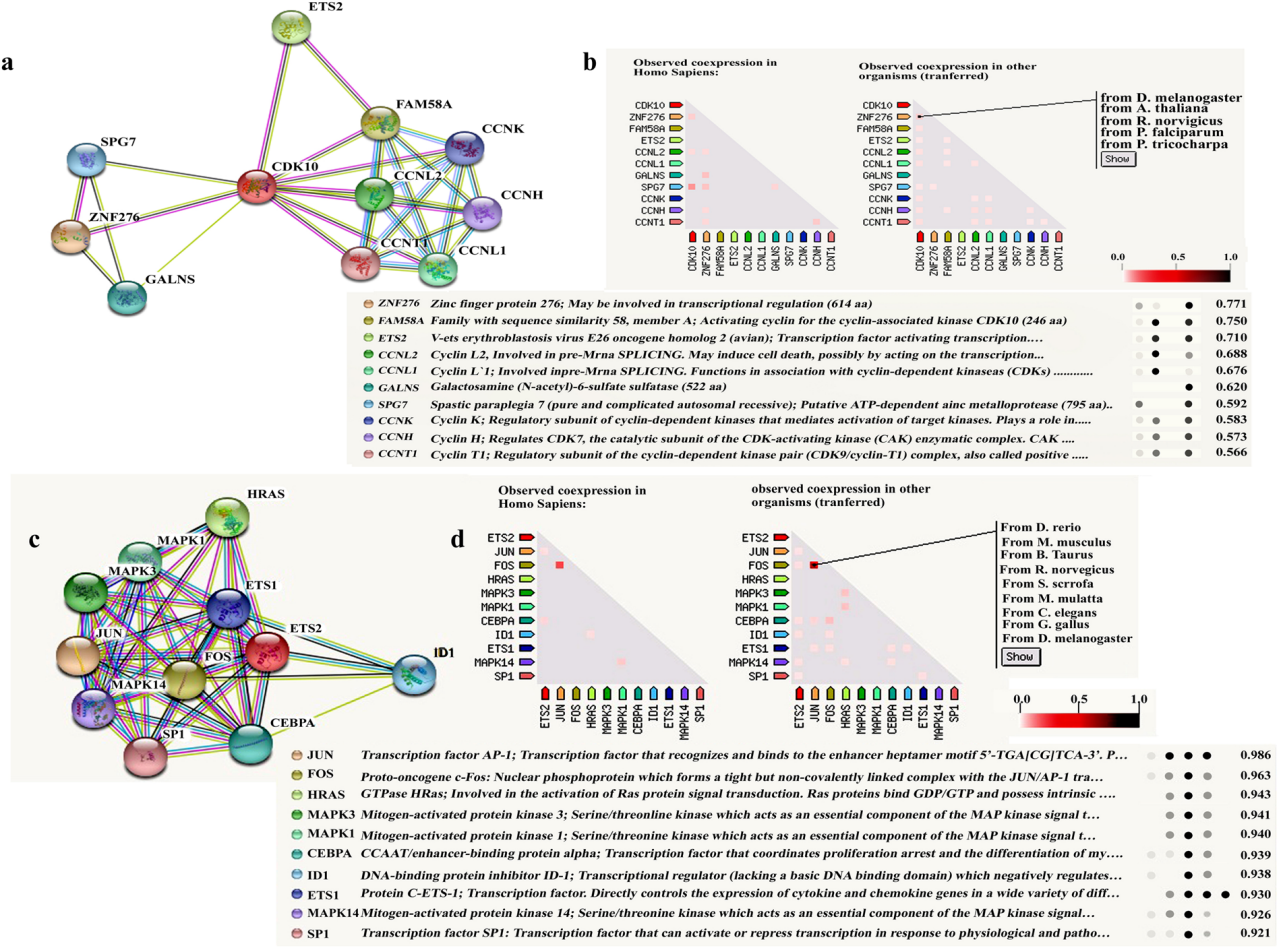
(**a**) Functional association network of cdk10: A total of 11 proteins including CDK10 were filtered into the PPI network complex using the STRING online database (http://string-db.org). **(b**) Co-expression predict its functional association with other partners. Each protein is represented as a node with edged interactions cdk10 cyclin dependent kinase 10; ZNF276 ZNF276 zin finger protein 276; FAM58A family with sequence similarity 58; ETS2 V-ets erythroblastosis virus E26 oncogene homolog 2 (avian); CCNL2 Cyclin L2; CCNL1 Cyclin L1; GALNS Galactosamine (Nacetyl)–sulfate sulfonate; SPG7 Spastic paraplegia 7 (pure and complicated autosomal recessive);CCNK,CyclinK;CCNH, CyclinH; CCNT1 CyclinT1. In the triangle-matrices above, the intensity of color indicates the level of confidence that two proteins are functionally associated, given the overall expression data in the organism. (**c**) Functional association network of ETS2: A total of 11 proteins including ETS2 were filtered into the PPI network complex using the STRING online database (http://string-db.org). (**d**) Co-expression predict its functional association with other partners. Each protein is represented as a node with edged interactions. ETS2: V-ets erythroblastosis virus E26 oncogene homolog 2 (avian); JUN Jun proto-oncogene; FOS FBJ murine osteosarcoma viral oncogene homolog; IDI Inhibitor of DNA binding 1, dominant negative helix-loop-helix protein;: HRAS v-Ha-ras Harvey rat sarcoma viral oncogene homolog; MAPK1 Mitogen-activated protein kinase 1; MAPK3 Mitogen-activated protein kinase 3; MAPK14 Mitogen-activated protein kinase 14; CSFIR Colony stimulating factor 1 receptor; ETS1:V-ets erythroblastosis virus E26 oncogene homolog 1 (avian); SP1Sp1transcriptionfactor.

The functional association network of the cdk10 protein was generated through protein-protein interaction network software STRING 8.3. The Search Tool for the Retrieval of Interacting Genes/Proteins (STRING) http://string-db.org is an online database containing known and predicted PPI networks. The protein-protein interaction patterns are helpful to provide a better understanding of the protein functional activities. A strong interaction of cdk10 with ETS2 and other important proteins is evident with high confidence score (>0.7). Each protein is represented as a node with edged interactions cdk10 cyclin dependent kinase 10;ZNF276 ZNF276 zin finger protein 276;FAM58A family with sequence similarity 58; ETS2 V-ets erythroblastosis virus E26 oncogene homolog 2 (avian); CCNL2 Cyclin L2; CCNL1 Cyclin L1; GALNS Galactosamine (Nacetyl)–sulfate sulfonate; SPG7 Spastic paraplegia 7 (pure and complicated autosomal recessive); CCNK, CyclinK; CCNH, CyclinH; CCNT1 CyclinT1; (Fig. 4a,b). A strong interaction of ETS2 with several other important proteins is evident with a high confidence score (>0.7). ETS2: V-ets erythroblastosis virus E26 oncogene homolog 2 (avian); JUN Jun proto-oncogene; FOS FBJ murine osteosarcoma viral oncogene homolog; IDI Inhibitor of DNA binding 1, dominant negative helix-loop-helix protein;: HRAS v-Ha-ras Harvey rat sarcoma viral oncogene homolog; MAPK1 Mitogen-activated protein kinase 1; MAPK3 Mitogen-activated protein kinase 3; MAPK14 Mitogen-activated protein kinase 14; CSFIR Colony stimulating factor 1 receptor; ETS1:V-ets erythroblastosis virus E26 oncogene homolog 1 (avian); SP1Sp1transcriptionfactor; (Fig. 4c,d).

### Western blot analysis

**Figure 5:**
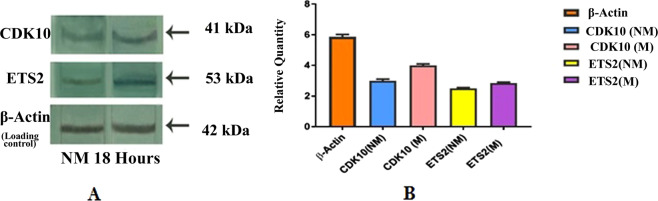
Western Blot analysis detected expression of cdk10 and ETS2 in HCEC: (**A)** The expression level of cdk10 and ETS2 at active hours of migration (18 hours) related to non-migrating sample with beta- Actin as loading control. lane 1 NM, non-migrating; lane 2, migrating at 18 hrs. **(B)** Quantification and intensity measurement of relative protein expression were analyzed by Quantity One software (Bio-Rad, USA). Histograms are generated using GraphPad Prism software (7.04).Values are expressed as ± SD, significance (P < 0.005) was calculated using one way ANOVA test statistically.

To validate the identification and interaction of cdk10 and ETS2 in corneal epithelial wound healing, western blot is performed which confirms the potential involvement of both the proteins in cellular migration and proliferation. The immune reactive band of cdk10 and ETS2 at 41 kDa and 53 kDa respectively while low expression of cdk10 was observed in non-migrating samples (Fig. 5A,B). However, densitometric analysis showed an increase in expression of cdk10 (17%) and ETS2 (6%) accurately reflects the contribution that each protein makes on the progression of wound healing.

### CO-Immunoprecipitation (Co-IP)

**Figure 6:**
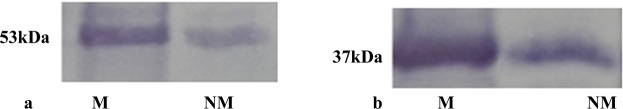
Co-immunoprecipitation was employed to validate the cdk10 and ETS2 interactions predicted by STRING. (**a**) An immunereactive band of cdk10 was detected at 41 KDa in (M) migrating (at 18 hours) and (NM) non-migrating samples. (**b**) The blot was stripped and probed with anti-ETS2 antibody showing band at 53 KDa in same samples.

In order to verify the interaction between cdk10 and ETS2, Co-IP combined with western blot analysis was used. cdk10 was detected in non-migrating while increased expressions were recognized at active phase of migration (18 hours). However, the blots were striped and probed with anti-ETS2 antibody in both migrating and non-migrating samples which shows that enhancement and activation during active cell migration (Fig. 6).

### Real Time qPCR

**Figure 7:**
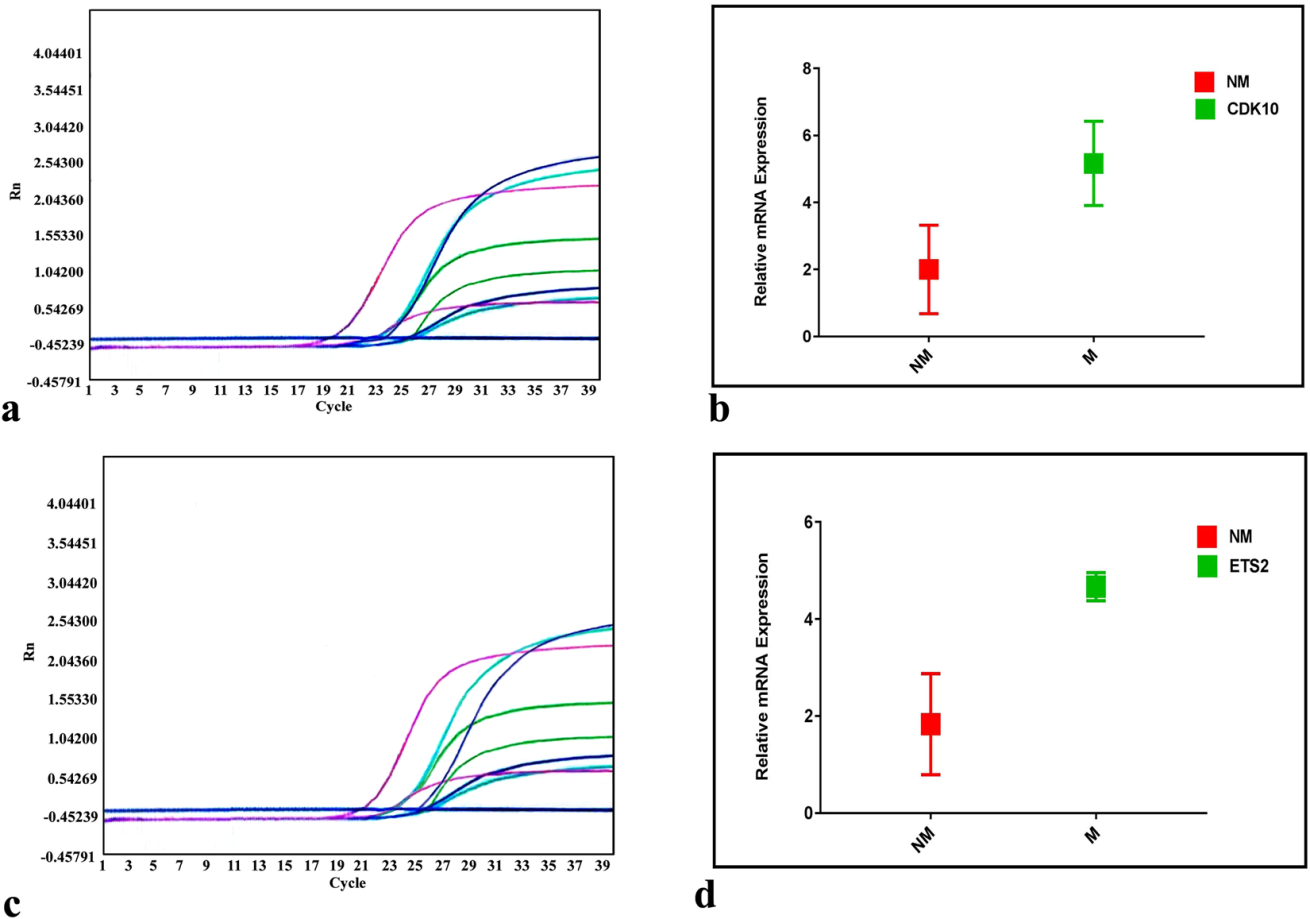
An amplification curve for cdk10 and ETS2 by real time qPCR. (**a**,**c**) Showing respective CT values for Non migrating (NM, a. light blue), and migrating (M, c. Pink), GAPDH (dark blue), and Negative control (green colour). (**b**,**d**) Relative mRNA expression of cdk10 and ETS2 in migrating (18 hours) post wounding was detected and validated by real-time PCR. Relative-change in gene expression compared to control non-migrating was calculated using the 2−ΔΔCT method as described in Materials and methods. All data were normalized against levels of GAPDH mRNA expression within the same sample. Error bars indicates ± SD, P < 0.005 after three individual experiments.

Reltative quantification of target genes cdk10 and ETS2 in human corneal epithelial cell migration at active hours were performed with quantitative real-time PCR (qPCR). Ct (Fig. 7a,c) and ΔCT values were measured and calculated with reference gene (GAPDH). Our results showed upregulation cdk10 and ETS2 gene expressions approximately 3 and 2 fold at active phase of migration repecteively as compared to non-migrating (Fig. 7b,d). Ct value is specific to gene of interest (SGI) and correspond to the number of cycles to reach a define threshold. ΔCt corresponds to the difference between Ct SGI and Ct of our reference sequence (RS), a house keeping gene (GABDH). ΔCt = CtSGI - CtRS.

Fold change was calculated by 2^**−**ΔΔ^CT. (Table 3).

**Table 3:** Fold change expression of cdk10 and ETS2 genes during non-migrating and migrating corneal epithelial cells.

Genes	ΔC_T_ mean *Non-migrating (NM)*	Δ C_T_ mean *Migrating (18* *hours)*	ΔΔ C_T_	2^−^ΔΔC_T_	P value
Cdk10	−2	−5.16	−3.16	6.33	0.0398*
ETS2	−1.8	−4.66	2.833	5.5	0.0105*

*p < 0.05 = statistically significant; CT, Threshold cycle.

### Effect of cdk10 and ETS2 on corneal epithelial wound healing

**Figure 8:**
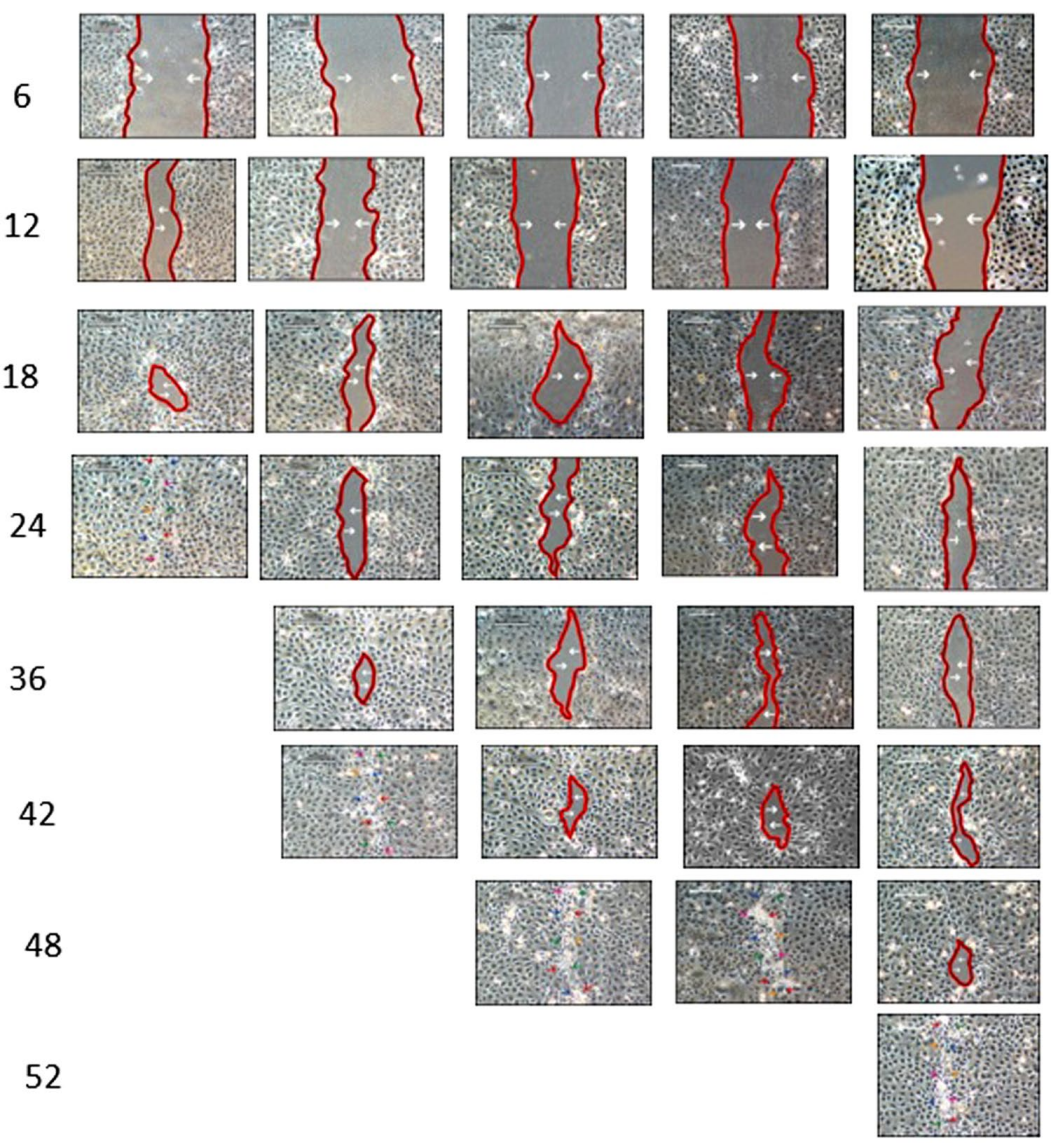
Delayed migration by anti-cdk10 and ETS2 antibodies. Cell migration was determined by a scratch assay. Confluent corneal epithelial cells were scratched and supplemented with 5 and 10 ug/ml cdk10 and ETS2 antobodies in serum free media then photographed using phase contrast microscopy at 6, 12, 18, 24, 36, 42, 48 and 52 post wounding.

**Figure 9:**
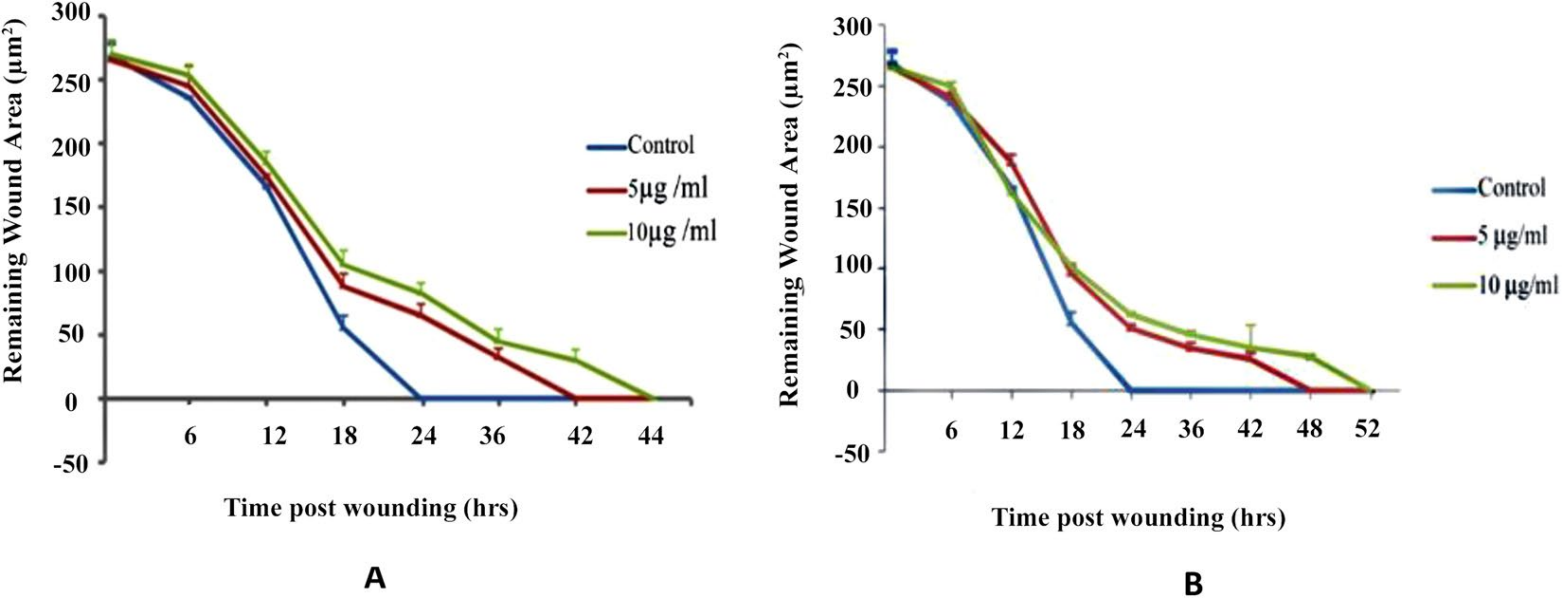
Effect of anti-cdk10 and anti-ETS2 antibodies on the rate of re-epithelialization of corneal wounds. Corneal re-epithelialization was observed in serum free media with and without (**A**) anti-cdk10 and (**B**) anti-ETS2 antibodies at 5 ug/ml and 10 ug/ml. Remaining wound area was measured at different time intervals (0–52 hrs) using Image J software. Bar indicates SDM. P < 0.0001.

To determine the effect of cdk10 and ETS2 on the cell migration activity of corneal epithelial cells, cells were cultured in serum free SHEM medium supplemented with two different concentrations (5 µg/ml & 10 µg/ml) of cdk10 and ETS2 (Fig. 8). We have found that exogenous anti-cdk10 and anti-ETS2 antibodies retard corneal epithelial wound healing at both concentrations, however they are most effective at higher concentration (10 ug/ml). At low concentration anti-cdk10 antibody delays the wound healing with wound closure at 42 hours while at higher concentration wound was closed at 48 hours as compared to control. In case of anti-ETS2 antibody at 5 ug/ml wound closure occurred at 42 hours compare to 10 ug/ml concentration to which wound was closed at 48 hours (Fig. 9A,B).

## Discussion

Corneal epithelial wound healing mechanism are the first aid in proper repair after injury and when these fail, wounds become chronic that could lead to permanent loss of vision. In some corneal disease, proper migration of epithelium across the denuded state is often impaired and considered as crucial aspects of healing in many defect. In these cases it is important to know the principal events in normal corneal healing proceses and the mechanism to restore corneal transparency. It is consistent with the already established old phenomenon, which says wound healing is a physiological process in which cell migration and proliferation related to cell division and cell cycle are stringently controlled by cell cycle-related proteins. However, little is known about the expression and the role of cyclin dependent kinases during corneal epithelial wound healing. Here, in this study we have estimated the cdk10 level of expression in active phase of cell migration and its association with ETS2 protein which has been confirmed by Co-immunoprecepitation, *in-silico* study and western blotting.

In the present study, the effects of cdk10 on cell migration activity were separately evaluated to establish more precisely the mechanism of cdk10. To the best of our knowledge, this is the first study showing association of transcription factor ETS2 with cdk10 protein during corneal epithelial wound healing and we also reports how alteration of their expression, could impact wound repair at both transcriptional and translational levels. Our expression analysis data obtained from *in-vitro* scratch wound healing model following complete re-epithelialization or closure of wound indicated a gradual and robust increase in cdk10 and ETS2 expression specifically at active phase of migration (at 18 hours) that coincided with the cell-proliferation phase of the wound repair process and revealed its role for the cellular processes. Furthermore the expression level of cdk10 also showed a decline by post wounding at 6 hours and at time of complete re-epithelization suggested their functions autonomously in the corneal healing. Similarily, Guen VJ and his group have detected an increased ETS2 expression level in cells derived from a STAR patient^17^. However, various studies have also supported our finding that cdk10 encodes two isoforms, each possess a different function within the cell cycle, one cdk isoforms interacts with the transcription factor ETS2, and modulating its transactivation activity, while the other is thought to have a role at the G2/M transition^11,18,19^. In addition, our *in-silico* and co-immunoprecepitation studies indicate a direct link between cdk10 and transcription factor ETS2 at active 18 hours of post wounding, therefore, being the first to describe an interrelation during corneal epithelial cell growth.

We also conducted STRING 8.3 generated patterns which demonstrated direct interaction of cdk10 and ETS2 proteins with other associated proteins.

This *in-silico* studies revealed that these interaction network has an essential role in regulation of phosphorylation of RNA, cell migration, cyclin dependent protein, nitrogen compound, cellular metabolic process and MAP kinase. Our results showed a number of significant biological function and shed light on the cdk10 and ETS2 regulation and contribute to the understanding of the underlying molecular mechanisms of corneal wound healing. Another potential clinical implications of our study are the effect of topical applications of cdk10 and ETS2 antibodies in *in-vitro* scratch assay that revealed significantly delayed migration in 10ug/ml concentration and delay wound closure, presumably a consequence of decreased cdk10 and ETS2 expression. Intrestingly, previous study supported our finding that cdk1 and cdk2 are activated only during cell cycle progression^19^ which is suppressed during closure of a small debridement wound^20^. In agreement with this study, our results showed that 10 ug/ml is an optimal concentration for delayed in cell migration compared to 5 ug/ml, however future studies are needed to conduct a dose dependent *in-vivo* experiments to clarify the role of cdk10 and ETS2 in proliferative activity during corneal wound healing. In contrast several lines of evidence provide insights into potential mechanism by which cdk10 act like a putative new tumor suppressor gene in multiple types of human cancers^21–24^. However, relatively little is known about its expression pattern, clinical relevance, and biological function in corneal wound healing. It is possible that cdk10 transiently upregulated by wounded epithelium might interact with ETS2; thus, it may facilitate healing of epithelial defects. We also hypothesize that the increased expressions of cdk10 and ETS2 at active hours may indicate increased cell migration, and may signal an increased supply of new cells for active proliferation. Previous study of our laboratory showed the interaction of albumin with fibronectin, argues strongly for a critical and major role for this protein in promoting the healing process in rabbit corneal organ culture model^25^.

In order to better understand the cellular response to wound healing, mRNA expressions of cdk10 and ETS2 were also analyzed which showed upregulation of cdk10 and ETS2 mRNA expression at active hours (18 hours) of cell migration which raises the possibility that they could have a potential role at transcriptional level in corneal epithelial migration. It is of intrest that in non-migrating corneal epithelial cells, no such increase in cdk10 and ETS2 proteins and mRNA were observed indicating that during the wound healing process there is a relief in the control of transcription of this protein.

Similarly Elegant studies by Gao *et al*. have been demonstrated that cdk5 regulates the migration of cultured corneal epithelial cells during *in-vitro* scrape-wound closure^14^. In addition, previous studies have observed ETS2 deregulation in many cancers^23,26^ and a less than *two-fold overexpression of ETS2 present severe cranial abnormalities and in apoptosis^27,28^. Similarily, studies have also reported that over expression of cdk5 has a similar effect on corneal debridement wound healing in transgenic mice^15^ suggested that it exerts its effects on corneal epithelial cell migration and wound closure through negative regulation of a cytoplasmic tyrosine kinase (Src). However, foetal tissues and bone marrow which have the highest mitotic index also showed average or low levels of cdk10 mRNA^19^ which suggested that expression of cdk10 may vary from tissue to tissue. While reduced cdk10 expression independently predicts a poor prognosis in patients with gastric cancer^29^ and highly expressed in colorectal cancer^30^.

In addition, a study conducted by Windpassinger C etal uncovered germline cdk10 mutations which is responsible for a growth-retardation syndrome and this stunted growth can be modeled in cdk10-deficient mice^31^. Surprisingly this cdk10 deficient mice showed high level of expression of ETS2 in the absence of cdk10. However, in our study expected consequence of enhanced ETS2 and cdk10 expressions at active phase of cell migration would be a decreased risk to develop certain types of cancers.

Finally, our findings collectively provide a significant insight into the understanding of cell cycle protein cdk10 and its association with transcription factor ETS2, in particular, its role in the control of cell division during active phase (18 hours) of corneal epithelial wound healing. Further studies are needed to know the association and mechanism of the action of cdk10 and ETS2 in promoting cell proliferation.

## Material and Methods

### Human corneal epithelial cell (HCEC) culture

SV-40 immortalized human corneal epithelial cell line were grown as monolayer to achieve the 80–90% confluency to form a confluent monolayer in cell culture as previously reported^32^. The Human corneal epithelial cell (HCEC), were purchased from American Type Culture Collection (ATCC, Rockville, MD, USA). Cells were plated in 75 cm^2^ cell culture flasks using supplement hormonal epithelial media (SHEM) containing Dulbecco’s modified Eagle’s medium DMEM/F12 1:1, 5%FBS, 5 μg/ml Insulin, 0.1 μg/ml Cholera toxin, 10 ng/ml hEGF and 0.5%DMSO and then maintained at 37 °C in a 5% CO2 and a 95% humidifed atmosphere. Culture medium was changed every 24 hours and the cells were harvested using 0.05% Trypsin-EDTA (Gibco BRL, CA, USA). A passage number of ≤5 was used in this study.

### Cell viability assay

We assessed the human corneal epithelial cell viablility using trypan blue stain according to the manufacturer’s protocol. Brefly, in harvested cell suspension (10 µl) equal volume of 0.4% trypan blue stain was added and cell counting was done using hemocytometer under inverted light microscope (NIKON, Japan). Viable cells are those excluded from the stain.

### *In-vitro* scratch wound healing assay

*In-vitro* wound healing scratch assay was performed using confluent monolayer corneal epithelial cells. Primary human corneal epithelial cells were grown to confluence on collagen coated plates. For migrating group of cells, a linear scratch a cell free area (abrasion 1 mm) was made using a sterile 200-lL pipette tip^33^ while unscratched cells (non-migrating) were used as control group. Immediately after the scratch at 0, 6, 12, 18, 24, 36, 42 and 48 hours images of the scratch area were captured using the Zeiss AxioCam MR digital camera on a Zeiss Axioskop2 light microscope (Carl Zeiss). The remaining wound area was measured using ImageJ software, provided in the public domain at http://rsb,info.nih.gov/ijnih.gov, Bethesda, MD.

### Protein extraction and quantification

Cells from non-migrating and migrating epithelia were harvested at 6, 12,18 and 24 hours in PBS buffer containing 6 M urea, 7 M thiourea, 4% CHAPS with protease inhibitor cocktail. The cells were lysed by sonication followed by centrifugation at 12000 × g for 15 minutes. The process was repeated twice, the supernatants was pooled and dialyzed to remove salts and detergent. Protein concentration was measured using bicinchoninic acid protein assay (Pierce™ BCA Protein Assay Kit, ThermoFisher Scientific) with BSA as standard.

### Two dimensional polyacrylamide gel electrophoresis (2-DE)

Quantified protein (100 µg) from non-migrating and migrating cells or harvested HCEC cells (different time intervals) were dissolved in rehydration buffer (7 M urea, 2 M thiourea, 4% CHAPS, 0.2% (v/v) Ampholyte 3–10, 15 mM DTT, bromophenol blue) and rehydrated overnight by using immobilized pH gradients (IPG) strips (11 cm pH 3–10 NL). Isoelectric focusing (IEF) was performed in a Bio-Rad system at 20 °C as follows: 500 V for 1 hour, 1000 V for 1 hour with gradual increase to 8000 V and kept constant for 32000 Vh and each sample was run in triplicate. Prior to second dimension, strips were equilibrated in 37.5 mM Tris-HCl (pH 8.8) containing 6 M urea, 2% (w/v) SDS, 20% (v/v) glycerol, and 0.5% DTT, and re-equilibrated in the same buffer containing 4.5% iodoacetamide replacing DTT, each for 20 minutes. For second dimension gels were run at constant 100 V for 18 hour. The gels were later silver stained and scanned in a desktop scanner.

The gel images were densitometrically analyzed with Delta 2DE imaging software 4.0 (Decodon, Greifswald, Germany) and merged to create a single master gel. The spots were marked and transferred to all images followed by normalization according to the total spot density. The quantity of each spot in a gel was normalized as a percentage of the total quantity of all spots.

The statistical analysis was performed with Delta 2DE incorporated statistics and Student’s t-test was applied to analyze the statistical variations and significance of protein expression. A value of p < 0.05 was considered statistically significant.

### Protein identification by MALDI-TOF

#### Mass spectrometry

Single protein spots were excised from the 2D gels and in-gel digested as described previously^16^. The tryptic fragments were analyzed by MALDI MS (voyger DE-PRO; Applied Biosystems) for sequence information. Samples for MALDI analysis were mixed in a 1:1 v/v ratio with a saturated CHCA solution in 50% ACN/0.1% TFA. A capillary voltage of 800–1000 V was applied together with a cone voltage of 40–45 V and collision energy of 4.2 eV. The samples aerosol was desolvated in a stream of nitrogen. Proteins were identified from peptide masses and amino acid sequences using MASCOT database.

#### Data processing

Protein identification was carried out using MASCOT 2.4 software (Matrix science, London, United Kingdom) identified against the UniProtKB with *Homo sapiens* species filter. The database was searched using trypsin as enzyme and iodoacetamide as cysteine blocking agent. Carbamidomethyl C was set as a fixed modification whereas up to two missed tryptic cleavages were allowed. Search tolerances were specified to 10 ppm for the precursor mass and 0.05 Da for-fragment masses, and FLEX-PC, ultraflex TOF/TOF as instrument type was used. Theoretical PI and molecular mass of individual protein spots were calculated by https://web.expasy.org/compute_pi/ while experimental data obtained from 2D gels analysis done by Melanie 9.0 software.

### *In Silico* analysis of the cdk10 by STRING

We have used search tool STRING 8.3 database (http://stringdb.org/) for protein–protein interaction (PPI) analysis and to explore biological association network among neighboring partner specifically for cdk10 and ETS2.

The basic interaction unit in STRING database, is the proteins ‘functional association’ that jointly contribute to the same functional process^34^.

### Validation of identified proteins by western blotting

Validation of the identified differentially expressed proteins was done by immunoblotting^35^. Briefly, extracted protein (20 µg) was loaded on 10% polyacrylamide gel and run at constant 100 V. The separated proteins were electrotransferred to PVDF membranes (Amersham GE, Munich, Germany) at 300 mA for 180 minutes on Wet electroblotting systems (Bio-Rad) that later blocked with 5% Bovine serum albumin (BSA) in Tris-Buffered Saline and Tween 20 (TBST) for 1 hour at room temperature (24 °C). After blocking, the blots were incubated separately with anti cdk10 and ETS2 antibody (ab58327, Abcam, USA), and anti β-Actin antibody (ab8227, Abcam, USA) (1:2000) overnight at 4 °C. After washing with TBST, goat anti mouse IgG HRP was applied (1:10,000) for 1 hour at room temperature. The blots were thoroughly washed and developed with TMB reagent and image was acquired and analyzed on Gel Documentation system (Bio-Rad, USA).

### Co-Immunoprecipitation

Immunoprecipitation was performed, using proteins extracted from active phase 18 hours post wounding (migrating) and non-migrating corneal epithelial cells as described above. Briefly, samples were precleared with 50 ml of protein A Sepharose (4 fast flow, GE Healthcare) by incubating at 4 °C for 1 hour and centrifuged. The supernatants were transferred into new tubes and incubated overnight at 4 °C with 10 mg of anti-cdk10 and 50 ml of protein A-agarose. The reaction mixtures were centrifuged, washed, and heated for 5 minutes at 95 °C in 20 ml Laemmli loading buffer. Samples were loaded on 10% gel and electrophoresis was performed. Separated proteins were electrophoretically transferred onto nitrocellulose as described elsewhere^35^. Nonspecific signal was blocked by overnight incubation of membrane in blocking buffer containing 5% nonfat dry milk in Tris-buffered saline Tween 20 (TBST). After washing with TBST, it was probed with anti cdk10 antibody 1:500 dilution at 4 °C for 1 hour. The blots were washed with TBST, incubated with secondary anti-rabbit IgG, and processed with ECL Western Blotting Detection System (GE Healthcare) and peroxidase activity was visualized according to manufacturer’s protocol. For ETS2 expression, the blot was stripped in a buffer containing 100 mM b-mercaptoethanol, 10% SDS and 62.5 mM Tris- HCL at 55 °C for 1 hour in a shaking water bath. It was probed with primary anti-ETS2 antibody (1:500 dilution) followed by a secondary anti-sheep IgG antibody using standard protocols of detection.

### Isolation of RNA and RT-qPCR

Total RNA was extracted from harvested cells after post wounding at 18 hours and non-migrating cells using mRNA extraction kit (VIOGENE, USA) according to the manufacturer’s protocol.

RNA yield was ascertained with Qubit assay kit (ThermoFisher Scientific) and to assess the integrity, total 3 µg RNA is used on a denaturing agarose gel stained with ethidium bromide (EtBr). We generated cDNA using 5 µg total RNA from each samples and relative expression of the cdk10 and ETS2 genes were determined using innuSCRIPT One Step RT_PCR SyGreen kit (ANALYTIK JENA). Briefly, the reaction conditions consisted of 0.5 µl of cDNA and 0.2 µM primers in a final volume of 10 µl of qPCR mix. Each cycle consisted of denaturation of 95 °C for 10 second, annealing at 60 °C for 20 second and extension at 72 °C for 1 minute.

The threshold cycle (CT) was used to estimate the amount of target mRNA. Ct values were collected for cdk10, ETS2 genes (genes of interest) were normalized to GABDH for each sample (Δ*C*_t_ = *C*_t_ gene of interest − *C*_t_ GAPDH) and for relative fold-change = 2−∆∆CT was used to quantify the amplified transcripts.

The primers used in this study were as follows: cdk10 (Forward, 5′ TGGACAAGGAGAAGGATG 3′; Reverse 5′ CTGCTCACAGTAACCCATC 3′), ETS2 (Forward, 5′ AGCGTCACCTACTGCTCT GTC 3′; Reverse 5′ CCGTTGCACATCCAGCAA 3′) and GABDH (Forward, 5′ ACCCACTCCTCCACCTTTGAC 3′; Reverse 5′ CTGTTGCTGTAGCCAAATTCG 3′). All samples were assayed in triplicate and the mean of the three experiments was used as the relative quantification value.

### Effect of cdk10 and ETS2 on monolayer migration assay in HCEC

Confluent monolayers of human HCEC, cultured in SHEM media as described above. At 75–80% confluency cells were starved in serum-free medium containing 5 μg/ml insulin for 24 hours and 1 mm wide wounds were created using micropipette. Cells were washed with medium to remove suspended cells and incubated in media containing (5 &10 µg/ml) of anti- cdk10 and anti-ETS2 antibodies and cells were incubated in a CO_2_ incubator. Three fields of each scratch area were photographed at various time points until the cells completely covered the cell-free area. The initial width of the wounds was similar in controls and treated monolayers. Monolayer wound healing was monitored 6-hourly at 6, 12, 18, 24, 36 and 42 hours. The remaining wound area was measured using ImageJ software, provided in the public domain at http://rsb,info.nih.gov/ijnih.gov, Bethesda, MD.
