# Author Correction: Association of Cyclin Dependent Kinase 10 and Transcription Factor 2 during Human Corneal Epithelial Wound Healing *in vitro* model

**DOI:** 10.1038/s41598-020-71045-3

**Published:** 2020-08-27

**Authors:** Meraj Zehra, Shamim Mushtaq, Syed Ghulam Musharraf, Rubina Ghani, Nikhat Ahmed

**Affiliations:** 1 Department of Research, Department of Biochemistry, Ziauddin University, Karachi, 75600 Pakistan; 2 Department of Biochemistry, University of Karachi, Karachi, 75500 Pakistan; 3 Dr. Panjwani Center for Molecular Medicine and Drug Research, International Center for Chemical and Biological Sciences, Karachi, Pakistan; 4 H.E.J. Research Institute of Chemistry, International Center for Chemical and Biological Sciences, University of Karachi, Karachi, 75270 Pakistan; 5 Department of Biochemistry, Jinnah Medical and Dental College, Karachi, 74800 Pakistan; 6 Department of Biochemistry, Department of Bioscience, Barette Hodgson University, Karachi, 74900 Pakistan

Correction to: *Scientific Reports* 10.1038/s41598-019-48092-6, published online 14 August 2019

In this Article, for Figure 8, the panels for ETS2 (10 µg) at 18hours and Cdk10 (5 µg) at 36 hrs are incorrect. A corrected figure appears below, with the conditions labelled.

**Figure 8:**
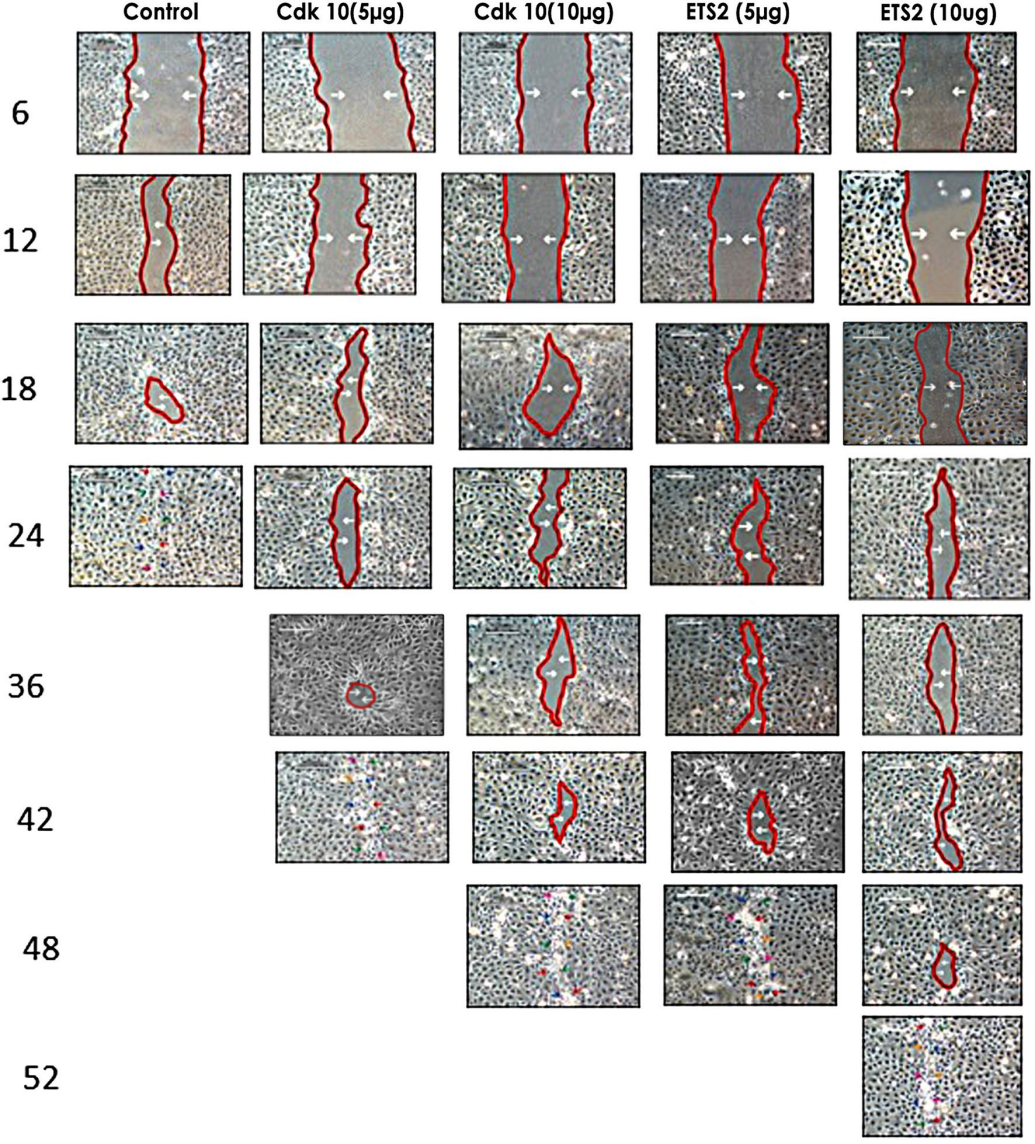
Delayed migration by anti-cdk10 and ETS2 antibodies. Cell migration was determined by a scratch assay. Confluent corneal epithelial cells were scratched and supplemented with 5 and 10 ug/ml cdk10 and ETS2 antibodies in serum free media then photographed using phase contrast microscopy at 6, 12, 18, 24, 36, 42, 48 and 52 post wounding

